# Outcomes in Pediatric Acute Lymphoblastic Leukemia—A Single-Center Romanian Experience

**DOI:** 10.3390/jcm9124052

**Published:** 2020-12-15

**Authors:** Mirabela-Smaranda Alecsa, Mihaela Moscalu, Laura-Mihaela Trandafir, Anca-Viorica Ivanov, Cristina Rusu, Ingrith-Crenguta Miron

**Affiliations:** 1 Department Mother and Child Care, Division of Neonatology, Grigore T. Popa University of Medicine and Pharmacy, 700115 Iasi, Romania; subotnicu_mirabela@yahoo.com (M.-S.A.); anca_vi@yahoo.com (A.-V.I.); abcrusu@gmail.com (C.R.); ingridmiron@hotmail.com (I.-C.M.); 2 Department of Pediatric Hematology and Oncology, Sf. Maria Children’s Emergency Hospital, 700309 Iasi, Romania; 3 Department of Preventive Medicine and Interdisciplinarity, Division of Informatics and Medical Statistics, Grigore T. Popa University of Medicine and Pharmacy, 700115 Iasi, Romania; 4 Department of Pediatrics, Sf. Maria Children’s Emergency Hospital, 700309 Iasi, Romania; 5 Department of Medical Genetics, Sf. Maria Children’s Emergency Hospital, 700309 Iasi, Romania

**Keywords:** childhood, acute lymphoblastic leukemia, outcomes, molecular biology

## Abstract

Background: This study evaluates the main (para)clinical aspects and outcomes in a group of Romanian children diagnosed with acute lymphoblastic leukemia (ALL), under the conditions of antileukemic treatment according to an adapted ALL IC Berlin–Frankfurt–Munster (BFM) 2002 protocol. Methods: We performed a retrospective single-center study of 125 children diagnosed with ALL between 2010 and 2016. Standard forms were used for data collection of variate clinical and paraclinical parameters. Results: The children were predominantly male (64.8%) and their median age at diagnosis was 5 years. A total of 107 patients were diagnosed with precursor B-cell acute lymphoblastic leukemia (BCP)-ALL and 18 with T-cell acute lymphoblastic leukemia T-ALL. Multiplex reverse transcription polymerase chain reaction RT-PCR assay for ETV6-RUNX1, BCR-ABL, E2A-PBX1, KMT2A-AFF1, and STIL-TAL1 fusion genes was performed in 111 patients. ETV6-RUNX1 translocation was detected in 18.9% of patients, while BCR-ABL1 and E2A-PBX1 rearrangements were seen in 2.7% and 3.6%, respectively. Complete remission at the end of induction phase was obtained in 89.6% of patients. The overall relapse rate was 11.2%, with 11 early and 3 late relapses. The 5-year overall survival rate in BCP-ALL was 81.6% and in T-ALL 71.4%. Conclusions: The 5-year overall and event-free survival rates in our study were slightly lower than those reported in developed countries, so the patients’ outcomes are encouraging.

## 1. Introduction

Among the different forms of cancer affecting children, the most commonly occurring is acute lymphoblastic leukemia (ALL). Fortunately, this illness is also one of the better understood and effective treatments are now available, tailored to different risk groups and enhanced by upgrades in supportive care [1,2,3,4]. However, universal accessibility is still a challenge and survival rates seem to echo economic and social disparities to a certain degree. In the more developed, high-income countries, survival rates have gone up to almost 90%, while in less prosperous countries, 30% of children are lost to the disease [1,5,6].

Whenever treatment can be promptly initiated and, importantly, adapted to the genetic profile of each patient, toxicity can be reduced and compliance maintained, resulting in optimal outcomes and remission [7,8]. Molecular genetics, in particular, has contributed substantially to the assessment of risks and prognoses depending on the course of action, thus enhancing customized care, maximized benefits, and lower relapse rates in pediatric ALL [9,10,11,12].

However, the international literature lists few studies from countries such as Romania, where pediatric patient families and oncologists must navigate less favorable social and economic conditions. Our study aims to appraise the clinical, hematological, and molecular features associated with ALL in a Romanian pediatric oncology center, as well as consider the context and its implications for the quality and effectiveness of care.

## 2. Materials and Methods

### 2.1. Study Design

This is a retrospective observational study using data from patients aged 1–17 who were newly diagnosed with ALL and admitted at the Oncology Department of the Sf. Maria Clinical Emergency Hospital for Children Iasi between January 2010 and December 2016. The study was approved by the Ethics Committees of the hospital and of the Grigore T Popa University of Medicine and Pharmacy Iasi. With 35 inpatient beds, this is the only pediatric oncology unit in northeastern Romania, servicing a total population about 4 million people, of which 1 million are under the age of 19 according to the most recent national census data available [13].

**Figure 1:**
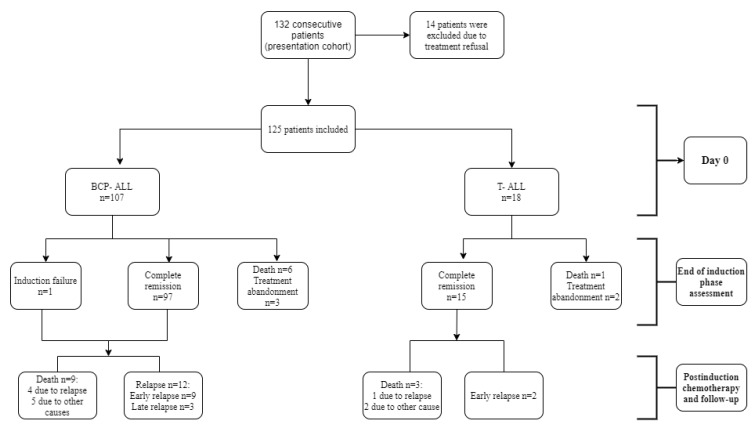
Flowchart of the studied cohort and treatment outcomes. BCP-ALL, precursor B-cell acute lymphoblastic leukemia; T-ALL, T-cell acute lymphoblastic leukemia.

From 1 January 2010 to 31 December 2016, 132 consecutive patients aged 1–17 presented at our department and were newly diagnosed with acute lymphoblastic leukemia. Fourteen patients were excluded after quitting treatment at our center (our knowledge of their treatment elsewhere and outcomes is anecdotal). Thus, a total of 125 ALL patients were eligible for our analysis (Figure 1).

### 2.2. Diagnosis

Peripheral blood samples, bone marrow (BM) aspirates, and lumbar punctures were collected at the time of diagnostic procedures and prior to any treatment. The ALL diagnosis was based primarily on the cytological examination of peripheral blood and bone marrow infiltration ≥25% blast cells (morphological and cytochemical evaluation of BM smears), and it was also confirmed through immunophenotypic analysis.

The most common translocations in precursor B-cell acute lymphoblastic leukemia BCP-ALL were also detected by means of molecular genetic techniques: ETV6-RUNX1, BCR-ABL1, KMT2A-AFF1, E2A-PBX1, and STIL-TAL1 translocation for T-cell acute lymphoblastic leukemia T-ALL. The assessment of minimal residual disease (MRD) was not possible because the necessary equipment was not available. Cerebrospinal fluid (CSF) was analyzed to determine the involvement of the central nervous system (CNS).

The patient information collected from medical records for the purposes of this study includes clinical and paraclinical variables such as age; gender; clinical status at admission; white blood cells (WBC) count; immunophenotyped; molecular biology; involvement of central nervous system; response to chemotherapy; relapse; last recorded follow-up; and, where applicable, death and cause of death. The patients whose legal guardians (parents or caregivers) refused the treatment, as well as those who abandoned the treatment protocol during first week, were excluded.

### 2.3. Immunophenotyping and Molecular Genetic Analysis

Immunophenotyping was carried out using a FacsCanto II Flow Cytometer (BD Biosciences, San Jose, CA, USA) and the classification criteria issued by the European Group for the Immunological Characterization of Leukemia (EGIL) [14].

Molecular tests were performed at diagnosis for the detection of four fusion genes: KMT2A-AFF1, ETV6-RUNX1, E2A-PBX-1, and BCR-ABL-p190. Ribonucleic acid RNA extraction was made from 2 × 10^7^ WBC. The cells were suspended in 0.5 mL of Guanidine Thiocyanate reagent (EZ-RNA Total RNA Isolation Kit, Biological Industries, Cromwell CT, USA) and stored at −80 °C until use. Total RNA was later isolated from the thawed cells according to the manufacturer’s instructions.

For reverse transcription, 4 μL of total RNA (with concentration of 500 ng/μL) was processed with the GoScript™ Reverse Transcription Kit (Promega, Madison, Wisconsin WI, USA). The complimentary DNA (cDNA) was diluted to a final volume of 100 μL, and 5 μL was amplified by polymerase chain reaction (PCR) for the detection of fusion gene transcripts.

RNA integrity was confirmed by PCR amplification of the mRNA of ABL gene, which is expressed ubiquitously in human hematopoietic cells.

For the amplifications, the GoTaq Green Master Mix (Promega, Madison, Wisconsin WI, USA) and 10 pmol of forward and reverse primers were used (Table 1) under strictly controlled conditions: 3 min at 95 °C, then 30 s at 95 °C, followed by 35 cycles of 30 s at the annealing temperature specific for each primer sets according to Table 1, 30 s at 72 °C, followed by 7 min at 72 °C. Cell lines were used as positive controls for the investigated fusion genes obtained from the Deutsche Sammlung von Mikroorganismen und Zellkulturen (DSMZ)-German Collection of Microorganisms and Cell Cultures, Department of Human and Animal Cell Cultures, Braunschweig, Germany and maintained in culture according to the recommendations from the DSMZ. The cell line used as positive control for STIL-TAL1 fusion gene was RPMI-8402 (DSMZ ACC-290).

The PCR products were migrated in a 2% agarose gel, and stained with ethidium bromide at 5 V/cm. The gel documentation was obtained with the UVP BioDoc-It System.

### 2.4. Risk Stratification and Treatment Protocol

The patients received intensive chemotherapy treatment according to a Berlin–Frankfurt–Munster (BFM) ALL 2002 protocol adapted for reasons outlined further down. The protocol entailed a 4-week course of chemotherapy with a cocktail of three specialized drugs (vincristine, anthracycline, and asparaginase) and a corticosteroid for the induction phase, followed by consolidation and reinduction therapy adapted to risk groups, and then maintenance therapy consisting of oral administration of 6-Mercaptopurine (6-MP) 50 mg/m^2^ daily and methotrexate (MTX) 20 mg/m^2^ weekly. Tyrosine kinase inhibitors (TKIs) were added for BCR-ABL positive patients.

Treatment response was evaluated based on absolute blast count in the peripheral blood (PB) on the eighth day of induction therapy: prednisone good responders (PGR) < 1000 blasts/μL and prednisone poor responders (PPR) ≥ 1000 blasts/μL, and based on bone marrow status on day 33 of induction treatment.

In order to evaluate the remission status, bone marrow punctures and evidence of extramedullary leukemia were assessed at day 33 of induction treatment. Patients who had <5% leukemic blast cells in their bone marrow and no signs of extramedullary disease on day 33 were considered to be in complete remission (CR).

The risk stratification of patients was done based on BM evaluation on day 15 and day 33 of induction therapy, absolute blast count in PB on day 8, and the presence of BCR-ABL or KMT2A-AFF1. Patients meeting at least one of the relevant criteria for high-risk (HR) were assigned to the high-risk group and were treated with the corresponding HR therapy branch. All the patients stratified into the other risk groups (standard and intermediate) were treated according to the intermediate risk therapy branch in order to mitigate the medical implications of episodes of insufficient or untimely supply of chemotherapy medication, which unfortunately sometimes occurs in Romania.

### 2.5. Conceptual Considerations

The study considered several key concepts as follows:

Event-free survival (EFS) was defined as the time from diagnosis to the date of the last follow-up indicating complete remission (CR) or the first significant event such as signs of resistance to chemotherapy (nonresponse), abandonment of treatment, relapse, or death from any cause.

Induction failure was defined as either morphological persistence of leukemic blasts in BM or extramedullary site(s) after the completion of the induction therapy.

Overall survival (OS) was defined as the period of time from diagnosis to the last follow-up or death.

Relapse was defined as the re-infiltration of bone marrow with more than 25% blast cells or the presence of blast cells in any other extramedullary site.

Moreover, note should be taken of the fact that patients’ follow-up was performed until 30 April 2020.

### 2.6. Statistical Analysis

The statistical analysis of the variables of interest was carried out in SPSS v.25 (IMB Corporation, Armonk, NY, USA). For continuous variables, we assessed the averages and standard deviation or the medians, depending on the normal distribution of the values. The comparisons between the statistical groups were done with the Mann–Whitney U test or the Kruskal Wallis test for continuous variables. The Levene test was used to assess the homogeneity of variances. For qualitative variables, we analyzed frequencies (absolute and relative %) and performed comparisons between groups based on the results of non-parametric tests (Yates and Chi-square). The Kaplan–Meier method was used to evaluate event-free survival (EFS) and overall survival (OS), and the log-rank test to make comparisons. The threshold for statistical significance (*p*) was set at *p* < 0.50.

## 3. Results

### 3.1. Epidemiologic Profiling

The median age at diagnosis was 5 years, and the most prevalent age group was 1–4 years (44.8%). Boys were predominant in the study group, with a male to female ratio of 1.84:1.

The characteristics of the patients with T-cell acute lymphoblastic leukemia (T-ALL) and of those with precursor B-cell acute lymphoblastic leukemia (BCP-ALL) are summarized in Table 2. The mean age at diagnosis was higher in T-ALL patients compared with BCP-ALL at *p* = 0.008. The WBC was found to be much higher than the normal range in T-ALL (median 70.61*10^3^/μL). The most frequent clinical features were hepatomegaly and splenomegaly in both BCP-ALL and T-ALL. The presence of initial CNS involvement was observed in only 4.8% of patients.

Risk assessment was possible in 122 patients; four of the seven patients who died before day 33 assessment had previously fulfilled the high-risk group criteria. A significant number of PPR and HR patients were noticed in the T-ALL group.

### 3.2. Immunophenotipyng and Molecular Findings

Immunophenotype data were available for all patients: 107/125 (85.6%) were precursor-B cell ALL and 18/125 (14.4%) were T-ALL. Multiplex RT-PCR assay to determine the presence of the most common ALL fusion genes (ETV6-RUNX1, BCR-ABL, E2A-PBX1, KMT2A-AFF1, and STIL-TAL1) was performed in 111 of 125 patients. The clinical, hematological, and prognostic characteristics of these subgroups were analyzed (Table 3).

The most common rearrangement was ETV6-RUNX1, found in 21 patients (18.9%) with a median age at diagnosis of 3 years. The patients with ETV6-RUNX1 had good prednisone response in 95.2% of cases and achieved complete remission in 85.7% of cases. Two patients presented an early relapse. A total of five deaths occurred in this subgroup: one due to relapse, three due to infection, and two due to hemorrhage.

We also detected four patients with E2A-PBX1 translocation. In this subgroup, the median age at diagnosis was 9.5 years. At the end of the induction phase, all these patients were in complete remission and one relapsed later during the maintenance phase of the protocol.

Three cases made up the BCR-ABLl subgroup; these patients’ median age upon diagnosis was 9 years. Although all three presented PGR, they were all lost to the disease: one relapsed 13 months after the initial diagnosis, one was a non-responder, and one succumbed to chemotoxicity.

In addition to the above, one patient presented both ETV6-RUNX1 and E2A-PBX1 translocations; he achieved complete remission and was subject to no other significant events during the study follow-up period.

KMT2A-AFF1 rearrangement was absent in all the patients.

The initial WBC was significant lower in the ETV6-RUNX1 subgroup (median of 8.32 *10^3^/μL) compared with the E2A-PBX1 subgroup (median of 89.96 *10^3^/μL), the BCR-ABL1 subgroup (median of 20.57 *10^3^/μL), and the patients without molecular modifications (median of 10.37 *10^3^/μL).

None of the patients diagnosed with T-ALL tested positive for the STIL-TAL1 fusion gene.

The patients’ initial WBC, their response to prednisone on day 8, their response to induction therapy on day 33, and the relapse cases across age groups as well as depending on the presence or absence of molecular modifications are summarized in Table 3.

### 3.3. Response to Chemotherapy, Relapse, and Treatment Outcomes

Of the 125 patients included in the study, 5 patients discontinued the treatment after achieving complete remission and were lost to follow up.

Death occured in 19 (15.2%) of patients. Seven patients (5.6%) unfortunately died before the assessment on day 33 and twelve patients (9.6%) died after achieving CR. Of these, 5 deaths were due to relapse and 7 were caused by chemotherapy toxicity or infections. Infections were the main cause of death (57.8%), followed by relapse (26.3%) (Table 4).

Regarding the response to prednisone, 93 patients diagnosed with BCP-ALL (86.9%) were good responders, and 12 patients diagnosed with T-ALL (66.6%) responded poorly (*p* = 0.030). At the end of the induction protocol, 112 (89.6%) patients achieved complete remission (Table 5).

The overall relapse rate across all the studied cases was 11.2%. The majority of relapsed patients (11/14, 78%) experienced an early relapse. One of them presented the BCR-ABL fusion gene and another had the ETV6-RUNX1 fusion gene. Three patients had a late relapse. The site of relapse was BM in nine patients (64%) and CNS in the other five (36%); there was no combined relapse identified in our patients. Moreover, 28.5% of relapsed patients were from the BCP-ALL high-risk group and relapsed early. Poor adherence to treatment was identified in three patients with an early relapse, two of which were from the HR group.

We evaluated the length of time from diagnosis to relapse, resistance to chemotherapy (non-response), recurrence, or death (event free survival, EFS). For the BCP-ALL patient group, the median EFS value was 56 months, while in the T-ALL patient group, the median EFS was of 40.5 months (Table 4). The median overall survival rates from diagnosis until death were the same. This may be because of the small number of T-ALL cases, but it is also worth mentioning that death has often been noticed to follow shortly after relapse, resistance to chemotherapy, or recurrence.

### 3.4. Survival

**Figure 2:**
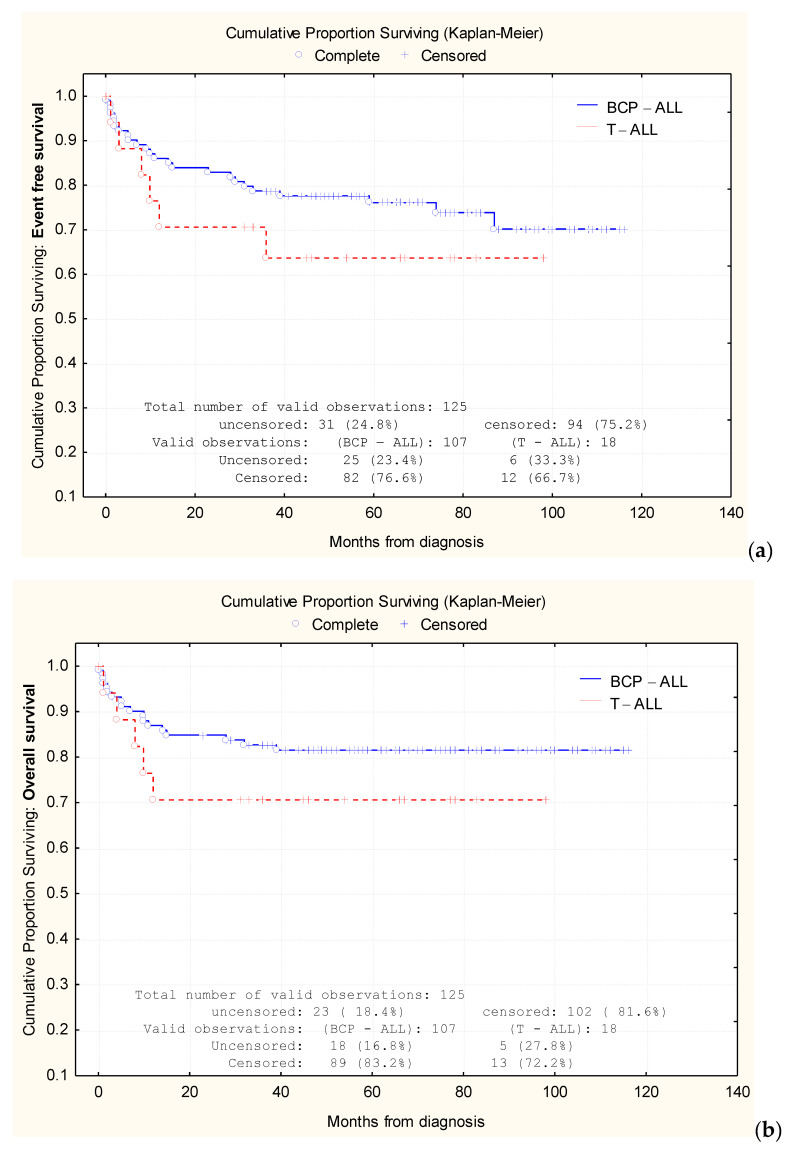
Comparative analysis of Kaplan–Meier curves between precursor B-cell acute lymphoblastic leukemia (BCP-ALL) cases and T-cell acute lymphoblastic leukemia (T-ALL) cases. Comparison of event-free survival (EFS) (**a**) rates and overall survival (OS) rates (**b**).

Based on the Kaplan–Meier analysis, we were able to compare the 1-year EFS rates between BCP-ALL cases (86.3%) and T-ALL cases (71.4%), as well as the 3-year EFS rates between the two groups (78.9% and 64.9%, respectively) and 5-year EFS rates: 76.1% in BCP-ALL vs. 64.9% in T-ALL (Figure 2a). Similarly, we analyzed the 1-year OS rates for BCP-ALL cases (87.2%) vs. T-ALL cases (71.4%), the 3-year OS rates (82.9% vs. 71.4%, respectively), and the 5-year OS rates (81.6% vs. 71.4% respectively) (Figure 2b).

According to our results and observations, risk stratification could have important prognostic value. Higher EFS rates at 90-month follow-up were observed in patients aged between 1 and 4 years (83.24%), in standard risk group (77.5%), in patients with WBC < 10 *10^3^/μL, with BCP-ALL, and without CNS involvement. The results indicate that WBC > 50 *10^3^/μL, high-risk group, older age, and rural residency influence EFS rates (Table 6). Table 6 provides the EFS for various parameters analyzed in the studied patients.

## 4. Discussion

In the absence of prompt and effective treatment, children with acute lymphoblastic leukemia can quickly succumb to the disease. Fortunately, complete remission is possible in a majority of cases, depending on medical and non-medical factors. In developed countries, cure rates are now as high as 80% to 90% [3,4]. By contrast, middle-income countries (MICs) report both more cases and lower cure rates, even when MICs have the necessary capabilities to adhere to and follow international therapeutic protocols. In these countries, specialized cancer care centers are fewer and farther between, chemotherapy medication can be inconsistently available, patients may delay presentation and treatment, compliance is more difficult to maintain, and related toxicity is more consequential and even lethal [8,9,11].

In Romania, the Berlin–Frankfurt–Munster (BFM) for pediatric ALL is the most used protocol. This protocol is known internationally to lead to complete remission in about 85–95% of pediatric ALL patients [11]. Although Romania has recently been upgraded to a high-income country, our data are from before this relabeling and reflect clinical, economic, and social circumstances that are commonplace in many parts of the world. For instance, while access to the latest diagnostic and therapeutic approaches have greatly improved, some methods remain largely unavailable (e.g., MRD testing). Moreover, the ongoing, uninterrupted provision of medication in adequate supply may prove challenging at times, depending on other, non-medical factors. Similarly, the array of supportive care arrangements known to enhance quality of life and improve survival is yet to be fully deployed.

Clinical and biological features of our patients seem to be consistent with other published data. High rates of pediatric ALL were found in the 1–4 age group, while the mean age of our patients was 5 years, the same as that in the ALL-BFM 2002 study [11]. The male to female ratio in our study was 1.84:1, higher than U.S. reported data (1.56:1) [16], Nordic countries [17], or Surveillance, Epidemiology, and End Results (SEER) database [18]. Similar male preponderance was reported in Pakistan (66.1%) and in some areas of India [19,20].

In our study, the fusion gene positivity occurred in 26% of all patients. The most prevalent molecular modification in our patients was ETV6-RUNX1; it was confirmed in 21 (18.9%) patients and had a significant prognostic role. The patients responded well to the treatment protocol.

In addition, the frequency of other fusion genes such as BCR-ABL1 and E2A-PBX1 was much lower in our patients and similar to that found by other published studies [21]. Both BCR-ABL1 and E2A-PBX1 had some adverse characteristics such as hyperleukocytosis and older age at diagnosis. In high-risk molecular modifications such as BCR-ABL1, CR was achieved in two of three cases; one of these patients suffered an ALL relapse 13 months after diagnosis and died, confirming the adverse prognostic role of this translocation in pediatric ALL.

Moreover, E2A-PBX1 is associated with a poor outcome, but this can be improved with more intensive chemotherapy [22]. All our E2A-PBX1 positive patients (3.6%) achieved complete remission on day 33; one of them had an early relapse. The frequency of E2A-PBX1 gene in our patients was similar to that other published studies reported in the United States [22,23], but much lower compared with Europe [24,25,26,27].

Interestingly, one patient presenting both ETV6-RUNX1 and E2A-PBX1 fusion genes achieved complete remission and did not require treatment in the 2 years that followed.

Relapse occurs in approximately 15–20% of children and is considered a major challenge in pediatric ALL survival [28]. Isolated bone marrow relapses occur in 75% of cases. The most common sites of isolated extramedullary relapse are the CNS and the testes, found in about 12% of relapsed patients [28,29]. ETV6-RUNX1 positive patients are associated with a high risk of late relapses and complete remission after second line therapy [28]. In our study, one patient with ETV6-RUNX1 translocation presented an early relapse and was lost one month later as a result of infectious causes.

T-cell acute lymphoblastic leukemia has been considered a negative prognostic factor in pediatric ALL, although the outlook has improved thanks to risk-adapted chemotherapy [30]. These patients are still at increased risk of induction failure, relapse, or isolated CNS relapse. The proportion of T-ALL patients (14.4%) in our study was higher than that reported in the BFM 2002 study (13.3%). In Central European countries as the Czech Republic, this ratio reached 17.3% [11] and, in the last four studies conducted at St Jude’s Children’s Research Hospital, 13.9–17.4% of children with ALL had T-ALL [30,31].

In our study, minimal residual disease (MRD) testing was unavailable. This is a noteworthy limitation in clinical practice in our center and the risk-group stratification criteria in the ALL-IC BFM 2002 protocol were specifically created as an alternative to MRD evaluation in countries where the methodology of PCR-based MRD testing is not implemented [32,33]. Fronkova et al. revealed that BFM 2002 morphology-based group risk stratification identified most of MRD high-risk patients, but was not reliable for the low-risk group (standard and intermediate) [32]. Since 2017, PCR and flow cytometry-based MRD assessment became available in our department and enabled us to evaluate the patients’ response to treatment.

In our professional opinion, the poorer outcomes in our study group were due to the incidence of high-risk leukemia (BCR-ABL1) and unfavorable factors such as increased WBC (>50.0 *10^3^/μL), T-cell immunophenotype, and older age (15–17 years).

The mortality in pediatric ALL depends not only on the disease itself, but also on the occurrence of complications such as infections and chemotherapy-related toxicities. Rubnitz et al. concluded that the main cause of death (80%) during the induction phase was related to infectious causes [34]. In our cohort, 57.8% of patients died of infectious causes. Other causes of death included chemotherapy-related toxicities or bleeding. Moreover, 5.6% of patients died before achieving complete remission, which is more than in developed countries, where the induction mortality rate is below 2% [35].

A study performed by EUROCARE that included pediatric patients diagnosed with ALL during 2000–2007 showed a 5-year survival rate in Eastern Europe, varying from 70% in Bulgaria to above 80% in Poland, compared with more developed countries where the 5-year survival far exceeded 80% in all countries. Romania, however, was not included in this research [36].

At the 5-year mark, the OS and EFS were 81.6% and 76.1%, respectively, for BCP-ALL and 71.4% and 64.9%, respectively, for T-ALL. The difference between OS and EFS might be attributed to the small number of T-ALL patients in our study (18 patients vs. 107 patients in the BCP-ALL group).

## 5. Conclusions

This study assesses the response to therapy and follow up of pediatric patients with ALL from NE Romania, taking into consideration their (para)clinical characteristics and genetic profile, as well as the fact that an adapted BFM-based treatment had to be used. Most notably, the patients’ survival rates were inferior to similar reports from high-income countries, but by a smaller than expected margin.

## Figures and Tables

**Table 1:** Sequences and annealing temperatures for primer sets used for polymerase chain reaction (PCR) amplifications, for fusion genes and the reference gene.

Fusion Gene	Forward Primer	Reverse Primer	Annealing Temperature
MLL-AF4	5′-CCGCCTCAGCCACCTAC-3′	5′-TGTCACTGAGCTGAAGGTCG-3′	65
ETV6-RUNX1	5′-TGCACCCTCTGATCCTGAAC-3′	5′-AACGCCTCGCTCATCTTGC-3′	
E2A-PBX1	5′-CACCAGCCTCATGCACAAC-3′	5′-TCGCAGGAGATTCATCACG-3′	
BCR-ABLp190	5′-GACTGCAGCTCCAATGAGAAC-3′	5′-GTTTGGGCTTCACACCATTCC-3′	
STIL-TAL1	5′-TCCCGCTCCTACCCTGCAA-3′	5′-CGCGCCCAGTTCGATGAC-3′	

van Dongen et al., 1999 [15].

**Table 2:** Clinical and biological characteristics of 125 patients diagnosed with acute lymphoblastic leukemia. T-ALL, T-cell acute lymphoblastic leukemia; BCP-ALL, precursor B-cell acute lymphoblastic leukemia; CNS, central nervous system.

Characteristics	Overall *n* = 125	BCP-ALL *n* = 107	T-ALL *n* = 18	*p*-Value
Age in years (mean ± SD) ^§^	6.78 ± 4.83	6.38 ± 4.77	9.17 ± 4.57	0.009 *
Age, median (range) **	5 (3; 11)	4 (3; 10)	8 (6; 13)	
age 1–4 years	56 (44.8%)	54 (50.5%)	2 (11.1%)	0.011 *
age 5–9 years	34 (27.2%)	26 (24.3%)	8 (44.4%)	
age 10–14 years	21 (16.8%)	15 (14%)	6 (33.3%)	
age 15–17 years	14 (11.2%)	12 (11.1%)	2 (11.1%)	
Gender (male/female) ^‡^	81/44 (64.8/35.2%)	69/38 (64.5/35.5%)	12/6 (66.7/33.3%)	0.857
White blood cells, median (range), (*10^3^/μL) ^†^	11.14 (3.59; 44.30)	8.32 (3.40; 32.82)	70.61 (14.00; 192.64)	0.001 *
<10.0	61 (48.8%)	57 (53.3%)	4 (22.2%)	0.003 *
10.0–50.0	33 (26.4%)	29 (27.1%)	4 (22.2%)	
>50.0	31(24.8%)	21 (19.6%)	10 (55.6%)	
Initial CNS involvement ^‡^ (absent/present)	119/6 (95.2/4.8%)	102/5 (95.3/4.7%)	17/1 (94.4/5.6%)	0.871
Initial mediastinal mass ^‡^ (absent/present)	114/11 (91.2/ 8.8%)	104/3 (97.2/2.8%)	10/8 (55.6/44.4%)	<0.001 *
Hepatomegaly ^‡^ (absent/present)	37/88 (29.6/70.4%)	35/72 (32.7/67.3%)	2/16 (11.1/88.9%)	0.044 *
Splenomegaly ^‡^ (absent/present)	38/87 (30.4/69.6%)	36/71 (33.6/66.4%)	2/16 (11.1/88.8%)	0.037 *
Prednisone response ^‡^ (PGR/PPR)	105/20 (84/16%)	93/14 (86.9/13.1%)	12/6 (66.7/33.3%)	0.030 *
Risk stratification (*n* = 122) ^†^ Standard/High	98/24 (80.3/ 19.7%)	87/17 (83.7/ 16.3%)	11/7 (61.1/ 38.9%)	0.022 *

§ Mann–Whitney U test; ^†^ Pearson Chi-square test; ^‡^ Yates Chi-square test; ** values presented as median (range: Q25–Q75); * marked effects are significant at *p* < 0.05; BCP-ALL—B-cell acute lymphoblastic leukemia; T-ALL—T-cell acute lymphoblastic leukemia; SD—standard deviation; PGR—prednisone good response; PPR—prednisone poor response; CNS—central nervous system.

**Table 3:** Molecular subgroups and other parameters at diagnosis, as well as during and after treatment.

Parameters *n* = 111	ETV6-RUNX1 *n* = 21	E2A-PBX1 *n* = 4	BCR-ABL *n* = 3	ETV6-RUNX1 and E2A-PBX1 *n* = 1	NONE *n* = 82	*p-*Value
% of total	21 (18.9%)	4 (3.6%)	3 (2.7%)	1 (0.9%)	82 (73.9%)	
Age, years ^^^ median (range) **	3(3–5)	9.5(4.5–13.5)	9(5–15)	3(3–3)	6(3–12)	0.031 *
Age 1–4 years ^†^	15 (71.4%)	1 (25%)	0	1 (100%)	31 (37.8%)	0.041 *
Age 5–9 years	5 (23.8%)	1 (25%)	2 (66.7%)	0 (0%)	25 (30.5%)	
Age 10–14 years	1 (4.7%)	1 (25%)	0 (0%)	0 (0%)	17 (20.7%)	
Age 15–17 years	0 (0%)	1 (25%)	1 (33.3%)	0 (0%)	9 (10.9%)	
Initial WBC (*10^3^/μL) ^^^ median (range)	8.32 (34.0–28.0)	89.96 (40.86–140.0)	20.57 (4.87–41.74)	140.00	10.37 (3.39–55.29)	0.320
<10.0	11 (52.4%)	0 (0%)	1 (33.3%)	0 (0%)	41 (50%)	0.246
10.0–50.0	5 (23.8%)	2 (50%)	2 (66.7%)	0 (0%)	19 (23.2%)	
>50.0	5 (23.8%)	2 (50%)	0 (0%)	1 (100%)	22 (26.8%)	
Response to Prednisone ^†^ (PGR/PPR)	20 (95.2%) 1 (4.8%)	3 (75%) 1 (25%)	3 (100%) 0 (0%)	0 (0%) 1 (100%)	65 (79.3%) 17 (20.7%)	0.033 *
Response to induction therapy ^†^ CR Induction Failure Unknown	18 (85.7%) 0 (0%) 3 (14.3%)	4 (100%) 0 (0%) 0 (0%)	2 (66.7%) 1 (33.3%) 0 (0%)	1 (100%) 0 (0%) 0 (0%)	73 (89%) 0 (0%) 9 (11%)	<0.001 *
Relapse ^†^ No Yes Unknown	16 (76.2%) 2 (9.5%) 3 (14.3%)	2 (50%) 1 (25%) 1 (25%)	1 (33.3%) 1 (33.3%) 1 (33.3%)	1 (100%) 0 (0%) 0 (0%)	58 (70.7%) 10 (12.2%) 14 (17.07%)	0.553

^^^ Kruskal–Wallis test; ^†^ Pearson Chi-square test; ** values presented as median (Q25–Q75); * marked effects are significant at *p* < 0.05; WBC—white blood cells; PGR—good prednisone response; PPR—poor prednisone response; CR—complete remission.

**Table 4:** Causes of death.

Cause of Death	Induction Therapy	After Complete Remission	Total Patients (%)
Infection	6	5	11 (57.8%)
Chemotherapy related toxicity	0	2	2 (10.5%)
Relapse-Progressive disease	0	5	5 (26.3%)
Bleeding	1	0	1 (5.2%)
Total	7	12	19 (100%)

**Table 5:** Response to chemotherapy, relapse, and outcome. Univariate analysis.

Parameters	BCP-ALL *n* = 107	T-ALL *n* = 18	Overall *n* = 125	*p-*Value
Response to Prednisone ^‡^ (PGR/PPR)	93/14 (86.9/13.1%)	12/6 (66.7/33.3%)	105/20 (84/16%)	0.030 *
Response to induction therapy ^†^ CR CR failure Unknown	97 (90.7%) 1 (0.93%) 9 (8.41%)	15 (83.3%) 0 (0%) 3 (16.7%)	112 (89.6%) 1 (0.8%) 12 (9.6%)	0.508
Relapse: (14 of 125 cases) ^‡^ Early Late	12 (11.2%) 9 (8.4%) 3(2.8%)	2 (1.9%) 2 (1.9%) 0 (0%)	14 (11.2%) 11 (8.8%) 3 (2.4%)	0.894
Death before CR ^‡^	6 (5.6%)	1 (5.6%)	7 (5.6%)	0.842
Death after CR	9 (8.4%)	3 (16.7%)	12 (9.6%)	
Event free survival rate ^§^ median (range) (months) 95% CI for median	56 (36‒74) 41‒67	40.5 (10.5‒42) 29.5‒41.5	52 (26‒59) 40.5‒56.5	0.327
Overall survival ^§^, median (range) (months) 95% CI for median	56 (38–75) 43‒66	40.5 (10.5–44) 29‒42.5	52 (21–63) 39.5‒58	0.278

^†^ Pearson Chi-square test; ^‡^ Yates Chi-square test; ^§^ Kaplan–Meier method—log-rank test; * marked effects are significant at *p* < 0.05; PGR—good prednisone response; PPR—poor prednisone response; CR—complete remission. 95% CI—95% confidence interval; T-ALL—T-cell acute lymphoblastic leukemia; BCP-ALL—precursor B-cell acute lymphoblastic leukemia.

**Table 6:** Event-free survival (EFS) in relation to other parameters.

Parameters (*n* = 125)	*n*	EFS *n* (%)	^†^ EFS: Kaplan–Meier Method Cumulative Proportion Surviving: 90 Months		*p-*Value
			Estimate	Std. Error	
Risk stratification	122	31 (25.4%)			
Standard	98	17 (17.4%)	77.5%	0.028	<0.001 *
High	24	14 (58.3%)	37.7%	0.031	
Age, years	125	31			
Age 1–4 years	56	8 (14.3%)	83.2%	0.057	0.109
Age 5–9 years	34	13 (38.2%)	54.7%	0.042	
Age 10–14 years	21	5 (23.8%)	71.9%	0.074	
Age 15–18 years	14	5 (35.7%)	46.4%	0.051	
Initial WBC (*10^3^/μL)	125	31			
<10.0	61	11 (18.1%)	72.6%	0.037	0.292
10.0–50.0	33	10 (30.3%)	69.1%	0.040	
>50.0	31	10 (32.3%	59.2%	0.041	
Gender	125				
Male	81	17 (20.9%)	75.6%	0.029	0.159
Female	44	14 (31.8%)	57.2%	0.036	
Immunophenotype	125				
Precursor B-cell	107	25 (23.4%)	70.8%	0.018	0.327
T-cell	18	6 (33.3%)	64.9%	0.046	
Residence	125				
Rural	81	24 (29.6%)	62.7%	0.022	0.113
Urban	44	7 (15.9%)	81.6%	0.049	
CNS involvement					
Yes	6	3 (50%)	50%	0.104	0.223
No	119	28 (23.5%)	71.1%	0.075	

^†^ Kaplan–Meier method—log-rank test; * marked effects are significant at *p* < 0.05; EFS—event-free survival; WBC—white blood cells; PGR—good prednisone response; PPR—poor prednisone response; CR—complete remission; CNS—central nervous system.
